# How Auditory Experience Differentially Influences the Function of Left and Right Superior Temporal Cortices

**DOI:** 10.1523/JNEUROSCI.0846-17.2017

**Published:** 2017-09-27

**Authors:** Tae Twomey, Dafydd Waters, Cathy J. Price, Samuel Evans, Mairéad MacSweeney

**Affiliations:** ^1^ESRC Deafness, Cognition and Language Research Centre, University College London, WC1H 0PD, United Kingdom,; ^2^Institute of Cognitive Neuroscience, University College London, WC1N 3AR, United Kingdom,; ^3^Wellcome Trust Centre for Neuroimaging, Institute of Neurology, University College London, WC1N 3BG, United Kingdom, and; ^4^Psychology Department, University of Westminster, 115 New Cavendish Street, London, W1W 6UW

**Keywords:** deaf, language, plasticity, sign language, superior temporal cortex, visuo-spatial working memory

## Abstract

To investigate how hearing status, sign language experience, and task demands influence functional responses in the human superior temporal cortices (STC) we collected fMRI data from deaf and hearing participants (male and female), who either acquired sign language early or late in life. Our stimuli in all tasks were pictures of objects. We varied the linguistic and visuospatial processing demands in three different tasks that involved decisions about (1) the sublexical (phonological) structure of the British Sign Language (BSL) signs for the objects, (2) the semantic category of the objects, and (3) the physical features of the objects.

Neuroimaging data revealed that in participants who were deaf from birth, STC showed increased activation during visual processing tasks. Importantly, this differed across hemispheres. Right STC was consistently activated regardless of the task whereas left STC was sensitive to task demands. Significant activation was detected in the left STC only for the BSL phonological task. This task, we argue, placed greater demands on visuospatial processing than the other two tasks. In hearing signers, enhanced activation was absent in both left and right STC during all three tasks. Lateralization analyses demonstrated that the effect of deafness was more task-dependent in the left than the right STC whereas it was more task-independent in the right than the left STC. These findings indicate how the absence of auditory input from birth leads to dissociable and altered functions of left and right STC in deaf participants.

**SIGNIFICANCE STATEMENT** Those born deaf can offer unique insights into neuroplasticity, in particular in regions of superior temporal cortex (STC) that primarily respond to auditory input in hearing people. Here we demonstrate that in those deaf from birth the left and the right STC have altered and dissociable functions. The right STC was activated regardless of demands on visual processing. In contrast, the left STC was sensitive to the demands of visuospatial processing. Furthermore, hearing signers, with the same sign language experience as the deaf participants, did not activate the STCs. Our data advance current understanding of neural plasticity by determining the differential effects that hearing status and task demands can have on left and right STC function.

## Introduction

The brain is capable of considerable experience-dependent plasticity. Unique insight into the extent of this plasticity in the human brain is provided by those born severely or profoundly deaf. A robust and replicated finding is that when those born congenitally deaf are processing visual stimuli, they show enhanced activation, relative to hearing participants, in regions of the superior temporal cortex (STC) that respond to auditory input in hearing people. The aim of the current study was to investigate how auditory experience influences the function of the left and right STC.

Prior studies have shown stronger activation in the right STC in deaf than hearing participants in response to a wide range of nonverbal visual stimuli such as moving dot arrays (Finney et al., 2001; Fine et al., 2005; Vachon et al., 2013), arrows (Ding et al., 2015), flashes (Bola et al., 2017), and static and moving sinusoidal gratings (Shiell et al., 2014). In contrast, in left STC enhanced activation in deaf compared with hearing participants appears to be highly stimulus and task-dependent. For example, it is observed in response to sign language stimuli during sign target detection (Capek et al., 2010; Cardin et al., 2013) and semantic anomaly detection even when sign language experience is matched across deaf and hearing groups (MacSweeney et al., 2002, 2004). However, it has not been observed during spoken language tasks on written words (Waters et al., 2007; Emmorey et al., 2013), pictures (MacSweeney et al., 2008, 2009), or speechreading (Capek et al., 2010; but see Capek et al., 2008) even though speechreading, like sign language, involves the perception of linguistically complex, moving visual stimuli.

Plausibly, the enhanced left STC activation in deaf participants in response to sign language could reflect the demands on visuospatial working memory that are made during sign language processing but not when performing speech-based tasks. In addition to the right STC activation, Ding et al. (2015) have also reported the contribution of the left STC to visuospatial working memory in deaf participants during a visuospatial working memory task for colored arrows (i.e., nonverbal visual stimuli). Importantly this left STC activation was observed only during the maintenance and recognition phases of the task, not during the encoding phase when the visual stimulus was present (for commentary, see MacSweeney and Cardin, 2015). This account can explain why Bola et al. (2017) also reported increases in the left (and right) STC activation in deaf participants performing a visual rhythm working-memory task involving sequences of flashes.

To dissociate sensory, visuospatial, semantic, and phonological processing in left and right STC, we engaged deaf and hearing signers in three different tasks in response to pictures of two objects. Visual imagery and visuospatial working memory were engaged during a British Sign Language (BSL) phonological judgment task (MacSweeney et al., 2008). This task required participants to decide whether the BSL signs for the two objects depicted shared a BSL phonological parameter (handshape or location), which are used to describe the sublexical structure of signs (Stokoe, 1960; Brentari, 1998; Sandler and Lillo-Martin, 2006b). In addition, the same participants were engaged in semantic and perceptual tasks that placed minimal demands on visual imagery and visuospatial working memory while keeping the stimulus presentation constant.

To dissociate auditory experience from sign language experience, and to examine any possible interactions between hearing and sign language experience, we included two groups of deaf participants who were either early or late sign language learners and two groups of hearing participants who were also either early or late sign language learners. In line with previous studies, we predicted greater activation in deaf than hearing participants in right STC, regardless of task. In contrast, in the left STC we expected task-specific effects of deafness, with a stronger effect on the BSL phonological task than the semantic or visual tasks.

## Materials and Methods

### 

#### 

##### Participants.

Sixty participants were scanned. All participants knew BSL. All had normal or corrected-to-normal vision and all gave informed, written consent to participate in the study, which was approved by the University College London Research Ethics Committee. One participant was excluded due to a data acquisition problem. A further 11 participants were excluded because of excessive head motion in the scanner (i.e., voxel size >3 mm in translation or the equivalent in rotation calculated with 65 mm as the cortical distance; Wilke, 2014). Thus, data from 48 participants were included in the analyses. All participants were right-handed (measured by the Edinburgh inventory; Oldfield, 1971) and without any known neurological abnormality.

Four participant groups were tested: (1) deaf native signers who learnt BSL from birth [henceforth DE (deaf early); *n* = 11 (male = 4)]; (2) deaf non-native signers who began to learn BSL aged 15 or older [henceforth DL (deaf late); *n* = 12 (male = 6)]; (3) hearing native signers who learnt BSL from birth [henceforth HE (hearing early); *n* = 13 (male = 1)]; (4) hearing non-native signers who began to learn BSL aged 15 or older [henceforth HL (hearing late); *n* = 12 (male = 5)]. The mean age of each of the groups was as follows: DE: 35.03 years (range: 26.11–59.10 years); DL: 39.06 years (range: 29.01–55.05 years); HE: 36.01 years (range: 20.03–60.00 years); HL: 41.10 years (range: 25.10–56.02 years). There were no significant age differences between groups (*F*_(3,44)_ = 1.168, *p* = 0.333, η^2^ = 0.074).

To facilitate group matching, participants were tested on the BSL grammaticality judgment task (Cormier et al., 2012), on performance IQ (PIQ; block design subtest of the WAIS-R), on reading attainment (Vernon-Warden, 1996) and on English vocabulary (shortened version of the Boston naming test; Kaplan et al., 1983). The BSL grammaticality judgment data were missing from two DE and one DL participants; the reading attainment data were missing from two HE and one DL participants; and the English vocabulary data were missing from one HL participant. There were no significant differences among the groups on the BSL grammaticality judgment task (*F*_(3,41)_ = 1.322, *p* = 0.280, η^2^ = 0.088), PIQ (*F*_(3,44)_ = 1.086, *p* = 0.365, η^2^ = 0.069) or English vocabulary (*F*_(3,43)_ = 1.363, *p* = 0.267, η^2^ = 0.087). However, there were group differences on reading attainment (*F*_(3,41)_ = 8.989, *p* < 0.001, η^2^ = 0.397) such that HL scored significantly better than HE (*t*_(21)_ = 3.433, *p* = 0.002, *d* = 1.433), DE (*t*_(21)_ = 4.610, *p* < 0.001, *d* = 1.924), and DL (*t*_(21)_ = 4.397, *p* < 0.001, *d* = 1.835). There were no significant differences in reading attainment between the HE, DE, and DL groups.

All deaf participants reported being born severely or profoundly deaf. Past audiogram data was available for only half of the participants (DE: 5/11; DL: 6/12). The mean hearing loss in the better ear for the DE participants was 91.2 dB; range: 81–105. The mean hearing loss in the DL group was 102.0 dB; range: 91–116. See Table 1 for a summary of participant characteristics. The use of hearing aids varied across deaf participants. The preferred language at the time of the experiment was BSL for all deaf participants except one. The details of hearing aid use in deaf participants, language experience when growing up and preferred language in adulthood are detailed in Table 2.

**Table 1.** Participant characteristics

	Mean age, year:month	Reading attainment, year:month	PIQ, centile	English vocabulary, max = 30	BSL grammaticality judgement, %	Hearing level in the better ear, dB
HE	36:01 (10:10)	17:06 (1:11)	84.4 (8.1)	28.2 (1.6)	79.9 (8.5)	N/A
(*n* = 13)	20:03–60:00	14:08–21:00	61.0–91.0	24.0–30.0	66.7–95.0	
HL	41:10 (8.08)	20:02 (1:10)	89.8 (9.6)	28.4 (1.6)	82.2 (6.3)	N/A
(*n* = 12)	25:10–56:02	15:08–22:00	63.0–98.0	26.0–30.0	73.3–90.0	
DE	35:03 (11:03)	16:07 (1:11)	89.6 (11.3)	27.5 (1.2)	85.3 (8.5)	91.2 (10.7)
(*n* = 11)	26:11–59:10	13:06–18:06	66.0–99.0	25.0–29.0	66.7–91.7	81.0–05.0
DL	39:06 (7:09)	16:06 (2:02)	90.9 (10.7)	27.1 (2.3)	84.8 (5.4)	102.0 (11.5)
(*n* = 12)	29:01–55:05	13:00–19:06	66.0–99.0	22.0–30.0	76.7–96.7	91.0–116.0

Mean [SD] and range of age, reading attainment, performance IQ, English vocabulary, BSL grammaticality judgement and audiogram data. Past audiogram data was available for only half of the participants (DE −5/11; DL 6/12). HE, Hearing Early; HL, Hearing Late; DE, Deaf Early; DL, Deaf Late.

**Table 2.** The use of hearing aids and the experience of language use in deaf participants

Participants	Use of hearing aids	Language	
		Used when growing up	Preferred
DE1	Data missing	Data missing	Data missing
DE2	Rarely	BSL/SSE	BSL
DE3	Every/all day	BSL/SSE/SpE	BSL
DE4	Data missing	Data missing	Data missing
DE5	In the past	BSL/SSE/SpE	BSL
DE6	Rarely	BSL	BSL
DE7	Never	BSL	BSL
DE8	Every/all day	BSL	BSL
DE9	Never	BSL	BSL
DE10	Data missing	Data missing	Data missing
DE11	Every/all day	BSL/SpE	BSL
DL1	In the past	SpE	BSL
DL2	Rarely	SpE	BSL
DL3	Never	SpE	BSL
DL4	In the past	SpE	BSL
DL5	Every/all day	SpE	BSL
DL6	Rarely	SpE	BSL
DL7	Sometimes	SpE	BSL
DL8	Never	SpE	BSL
DL9	Data missing	Data missing	Data missing
DL10	Every/all day	SSE/SpE	BSL
DL11	Every/all day	SpE	SpE
DL12	Every/all day	SpE	BSL

Abbreviations: BSL = British Sign Language, SSE = sign supported English, SpE = spoken English.

##### Experimental design.

Two between-subject factors were included: hearing status (deaf vs hearing) and age of sign language acquisition (age of acquisition: early vs late). In addition, a within-subject factor, task, was included with three levels (BSL phonological, semantic, visual judgment). This resulted in a balanced, 2 × 2 × 3 (hearing status × age of acquisition × task) factorial design.

##### Stimuli and task.

The stimuli consisted of 200 pictures which were recombined to form 300 different picture pairs. Three picture pair sets were established such that 100 pairs were used in each of the three tasks: phonological, semantic, and visual judgment. Within each picture set, 50 pairs were established to form “yes” trials and 50 to form “no” trials. Overall this design ensured that the same pictures were used across all three tasks. All 200 pictures were used in the phonological and semantic tasks, whereas only 150 of the pictures were used in the visual task due to the nature of the “same picture?” task (see Visual task).

Of the 200 pictures, 194 were black and white line drawings depicting high-familiarity nouns, of which all but one (“dream”) was concrete. The remaining six pictures were colored squares representing color names. Half of the pictures were from the Snodgrass and Vanderwart (1980) normed picture set. The other half was sourced from a range of picture-naming projects and were selected or adapted to match the visual characteristics of the Snodgrass and Vanderwart (1980) set.

##### Phonological judgment task.

Twenty-five picture pairs were established in which the BSL label for the picture overlapped in handshape and 25 which overlapped in hand location. These are two of the phonological parameters of signed languages (Sandler and Lillo-Martin, 2006a). A further 50 picture pairs were established as “no” trials in which the BSL labels did not overlap in any phonological parameter and the items were not semantically related.

##### Semantic judgment task.

The 200 picture stimuli were recombined to form 50 category-related pairs (e.g., “pear–banana”, “drum–guitar”, “sun–moon”) and 50 unrelated pairs. These stimuli were piloted with 15 hearing native speakers of English. Only pairs in which 12 or more of the pilot participants reported a category relationship were used as “yes” stimuli in the fMRI study. Similarly, “no” trials were only used if a minimum of 14 of 15 pilot participants agreed that the pictures were unrelated.

##### Visual task.

**Figure 1.**
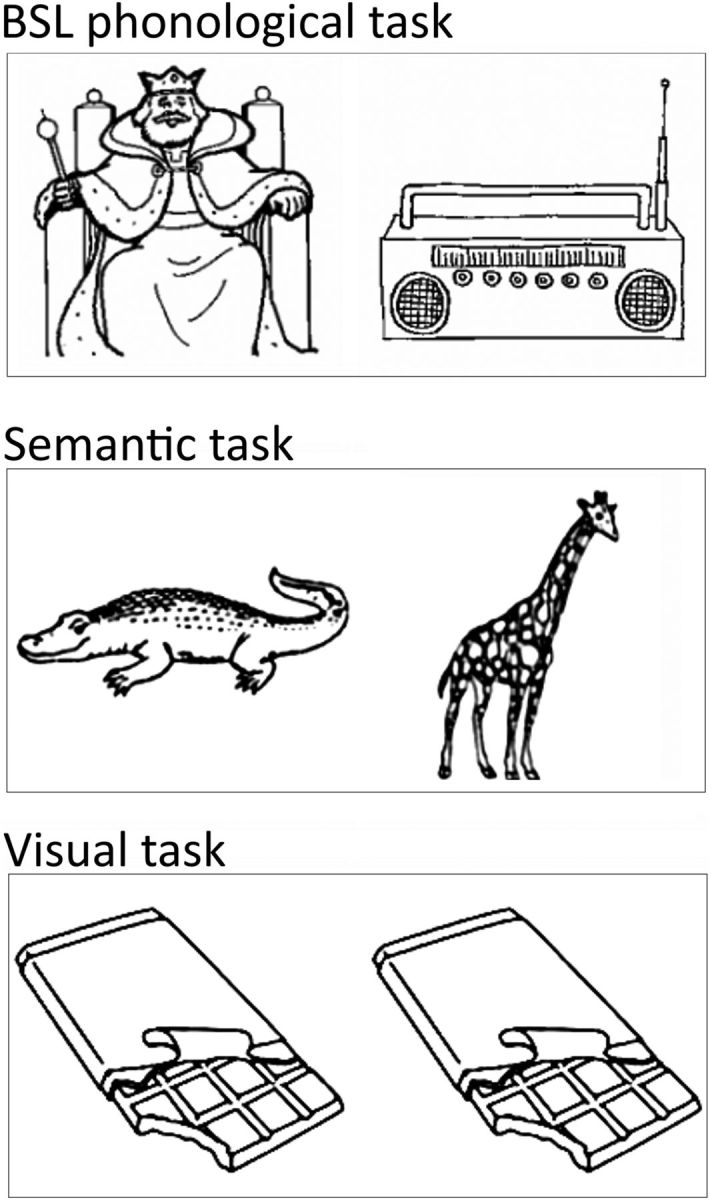
Stimulus examples. Top, BSL phonological task “Same handshape?” Middle, Semantic task “Same category?” Bottom, Visual task “Same picture?”

In the visual matching (same?) condition, 50 of 200 pictures appeared in 50 same-picture pairs (e.g., “sun–sun”) and 100 appeared in 50 different picture pairs (e.g., sun–pear). Examples of the stimuli are shown in Figure 1.

Due to lexical variation in BSL (Schembri et al., 2010), it was important to show participants all experimental pictures before the fMRI experiment to ensure that they used the desired BSL label, to facilitate the BSL phonological task. For each participant, there were only a few pictures where it was necessary to ask participants to base their decisions on signs that, although part of the BSL lexicon, were not the signs they usually used for the item.

##### Procedure.

Participants performed three judgment tasks: BSL phonological, semantic, and visual. In the BSL phonological task, participants were required to press a button when the BSL labels for the two pictures shared a sign phonological parameter. In separate blocks participants were required to detect shared handshape or shared location. In the current study, data are combined to form the “BSL phonological judgment” condition. The data contrasting handshape and location decisions will be reported separately. In the semantic task, participants were required to press a button when the picture pairs came from the same category (e.g., elephant/donkey). In the visual task participants judged whether the pictures presented were the same or different.

For all participants, the right index finger was used to respond to “yes” trials. “No” trials did not require a response. Half the trials in each condition were “yes” trials and half were “no” trials. Participants practiced the tasks, on stimuli not presented in the scanner, immediately before the fMRI experiment.

Each participant completed four fMRI runs (7 min each). Each run consisted of 15 × 21 s blocks of which five were BSL phonological decision blocks, five were semantic decision blocks and five were visual matching blocks. The order of presentation of conditions was pseudorandomized across runs. Each block began with a 1 s printed English task prompt (either “handshape?” or “location?” for the BSL phonological decision, “related?” for the semantic decision, or “same?” for the visual decision). This was followed by five picture-pair presentations, each with a 3.5 s exposure duration and an interstimulus interval of 500 ms. Task blocks were separated by baseline blocks of crosshair fixation: 13 × 6 s blocks; and two longer 13.5 s fixation blocks positioned in the middle and toward the end of the run. Stimuli were projected onto a screen positioned at the top of the scanner bore. Participants viewed the stimuli via a mirror placed on the MRI head coil.

##### MRI acquisition.

Anatomical and functional images were acquired from all participants using a Siemens 1.5-T Sonata scanner. Anatomical T1-weighted images were acquired using a 3-D MDEFT (modified driven equilibrium Fourier transform) sequence. One-hundred seventy-six sagittal partitions with an image matrix of 256 × 224 and a final resolution of 1 mm^3^ were acquired [repetition time (TR): 12.24 ms; echo time (TE): 3.5 ms; inversion time (TI): 530 ms]. Structural scans indicated that our participants were free from gross neurological abnormalities.

Functional T2*-weighted echo-planar images with BOLD contrast comprised 38 axial slices of 2 mm thickness (1 mm gap), with 3 × 3 mm in-plane resolution. One-hundred thirty-four volumes were acquired per run (TR: 3.42 s; TE: 50 ms; flip angle = 90°). TR and stimulus onset asynchrony were mismatched, allowing for distributed sampling of slice acquisition across the experiment (Veltman et al., 2002), which obviates the need for explicit “jittering”. To avoid Nyquist ghost artifacts, a generalized (trajectory-based) reconstruction algorithm was used for data processing. After reconstruction, the first six volumes of each session were discarded to ensure tissue steady-state magnetization.

##### Statistical analysis.

Behavioral data were analyzed in a 2 × 2 × 3 ANOVA with hearing status (deaf, hearing), the age of BSL acquisition (early, late) as between-subject factors and task (BSL phonological, semantic, visual) as a within-subject factor. The *d′* scores, accuracy and reaction times (RTs) were the dependent measures. Where Mauchly's test indicated significant non-sphericity in the data, a Greenhouse–Geisser correction was applied. When there was a main effect of task or interaction effects with task, planned comparisons were performed using paired *t* tests to evaluate differences between: (1) the BSL phonological and the semantic tasks, (2) the semantic and the visual tasks, and (3) the BSL phonological and the visual tasks. For the calculation of the *d′* scores, corrections of ±0.01 were made because some subjects had the hit rate of 1 and/or the false alarm rate of 0. RTs were measured for go trials only and were recorded from the onset of the stimulus. Anticipatory responses (<200 ms) were trimmed (*n* = 9; 0.05% of all the trials across participants).

The imaging data were processed using SPM12 (Wellcome Trust Centre for Neuroimaging, London UK; http://www.fil.ion.ucl.ac.uk/spm/). All functional volumes were spatially realigned and unwarped to adjust for minor distortions in the B0 field due to head movement (Andersson et al., 2001). All functional images were normalized to the Montreal Neurological Institute (MNI) space (maintaining the original 3 × 3×3 mm resolution). Functional images were then smoothed using an isotropic 6 mm full-width at half-maximum Gaussian kernel.

First-level fixed-effects analyses were based on a least-squares regression analysis using the general linear model in each voxel across the whole brain. Low-frequency noise and signal drift were removed from the time series in each voxel with high-pass filtering (1/128 Hz cutoff). Residual temporal autocorrelations were approximated by an AR(1) model and removed. At the first level, the onsets of stimuli (3.5 s) were modeled as epoch-related responses (for the exact duration of the stimuli) and convolved with a canonical hemodynamic response function. Correct trials for each of the three conditions over four sessions and the errors were modeled separately. Button press manual responses were modeled as event-related responses and convolved with a canonical hemodynamic response function. Fixation was not modeled and served as an implicit baseline. The contrasts of interest were each experimental condition (BSL phonological, semantic, and visual) relative to fixation, averaged over sessions.

At the second-level, a random-effects analysis included the contrast images for the three task conditions relative to fixation (within-subject) for each of the four (2 × 2) groups (between-subject), resulting in 2 × 2 × 3 ANOVA with hearing status (deaf, hearing), the age of BSL acquisition (early, late) as between-subject factors and task (BSL phonological, semantic, visual) as a within-subject factor with a correction for non-sphericity. The RTs, which may have contributed to the task effects, were not included in the imaging analyses because we were interested in the task difference.

We identified the effects in the left STC and the right STC separately. We first identified the effects of task modulation. Given the stepwise increase on the linguistic task demands, we specifically looked for the BSL phonological task > the semantic task; and the semantic task > the visual task. We then established whether deaf signers activated more than the hearing signers across tasks (i.e., the effect of deafness). Finally, we identified whether the effect of deafness was dependent on task and on age of BSL acquisition. We report activation as significant at voxel-level inference of *p* < 0.05, familywise error corrected for multiple comparisons at the whole-brain level (*Z* > 4.76). For effects within the left or right STC, we also report activation at an uncorrected level of *p* < 0.001 because we had a priori hypotheses regarding the function of these regions.

Lateralization was assessed using the bootstrapping procedure implemented within the LI toolbox (Wilke and Schmithorst, 2006; Wilke and Lidzba, 2007) in SPM. This is a robust tool that deals with the threshold dependency of assessing laterality from neuroimaging data (Bradshaw et al., 2017). We assessed lateralization for a main effect of group and interactions of group and tasks. The contrasts used were as follows: (1) deaf > hearing, (2) deaf > hearing by phonological task > semantic task, and (3) deaf > hearing by phonological task > visual task. Ten-thousand lateralization indices (LIs) were calculated from 100 bootstrapped resamples of voxel values in each hemisphere, at multiple thresholds. This analysis does not require a fixed threshold or correction for multiple comparisons because it is based on a bootstrapping procedure. Resulting LIs were plotted and the weighted mean, which gives greater weighting to higher thresholds, was calculated. A built-in temporal mask, which covers the entire temporal cortices, was selected as an inclusive mask. No exclusion mask was used. Analyses were conducted without clustering or variance weighting. Weighted laterality values ≥0.2 (left) or ≤−0.2 (right) indicate significant lateralization (Wilke and Schmithorst, 2006; Wilke et al., 2006; Lebel and Beaulieu, 2009; Lidzba et al., 2011; Badcock et al., 2012; Nagel et al., 2013; Pahs et al., 2013; Gelinas et al., 2014; Norrelgen et al., 2015; Evans et al., 2016). We also report the trimmed mean, which is calculated from the central 50% of all the LIs, for completeness.

## Results

### Behavioral data

The *d′* scores showed that there was a significant difference in response sensitivity as a function of tasks (*F*_(2,88)_ = 397.189, *p* < 0.001, η^2^ = 0.900). Planned *t* tests confirmed that *d′* for the BSL phonological task was significantly lower than the semantic task (*t*_(47)_ = 20.386, *p* < 0.001, *d* = 2.943) and the visual task (*t*_(47)_ = 26.924, *p* < 0.001, *d* = 3.885). In addition, *d′* for the semantic task was significantly lower than the visual task (*t*_(47)_ = 7.334, *p* < 0.001, *d* = 1.059). However, response sensitivity did not differ across hearing status (*F*_(1,44)_ = 0.665, *p* = 0.419, η^2^ = 0.015) or age of acquisition (*F*_(1,44)_ = 0.137, *p* = 0.713, η^2^ = 0.003) and the interaction of these two factors was not significant (*F*_(1,44)_ = 3.243, *p* = 0.079, η^2^ = 0.069). Other interactions were also nonsignificant (all *p* > 0.267).

**Figure 2.**
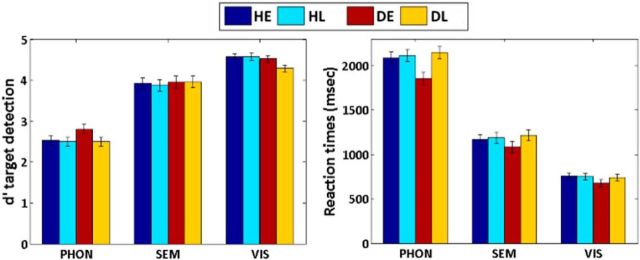
Behavioral results. Left, Response sensitivity (*d′*). Right, RTs (ms). Both show a main effect of task, and a significant task by age of acquisition interaction on the RTs only. PHON, BSL phonological task; SEM, semantic task; VIS, visual task.

A main effect of task was also significant for RTs (*F*_(1.559, 68.601)_ = 1530.809, *p* < 0.001, η^2^ = 0.972). The RTs were longer for the BSL phonological task than the semantic task (*t*_(47)_ = 34.920, *p* < 0.001, *d* = 5.042) and the visual task (*t*_(47)_ = 42.766, *p* < 0.001, *d* = 6.174) and for the semantic task than the visual task (*t*_(47)_ = 24.457, *p* < 0.001. *d* = 3.532). There were no main effects of hearing status (*F*_(1,44)_ = 1.362, *p* = 0.249, η^2^ = 0.030) or age of acquisition (*F*_(1,44)_ = 3.205, *p* = 0.080, η^2^ = 0.068). In the RT data however, there was a significant task × age of acquisition interaction (*F*_(1.56,68.60)_ = 3.828, *p* = 0.036, η^2^ = 0.080). *Post hoc t* tests confirmed that the participants who learnt BSL late (HL and DL) were significantly slower than those who learnt BSL early (HE and DE) on the BSL phonological task (2129.92 vs 1979.25, *t*_(46)_ = 2.136, *p* = 0.038, *d* = 0.617) but not on the semantic (1201.17 vs 1127.75, *t*_(46)_ = 1.227, *p* = 0.226, *d* = 0.354) or the visual tasks (744.38 vs 720.33, *t*_(46)_ = 0.637, *p* = 0.527, *d* = 0.184). The behavioral data are illustrated in Figure 2. Although Figure 2 suggests that this interaction might be driven by the deaf participants, there was no significant three-way interaction (*F*_(1.559,68.601)_ = 2.343, *p* = 0.116, η^2^ = 0.051). The interaction of hearing status and age of acquisition was also not significant (*F*_(1,44)_ = 2.381, *p* = 0.130, η^2^ = 0.051).

In summary, the behavioral data suggest that the BSL phonological task was more demanding than the semantic task, which in turn was more demanding than the visual task. Moreover, the effect of learning BSL late was evident in reaction times during the BSL phonological task only. There was no effect of hearing status on behavioral performance on the tasks or interaction between hearing status and any other factors.

### fMRI data

#### Left STC

**Figure 3.**
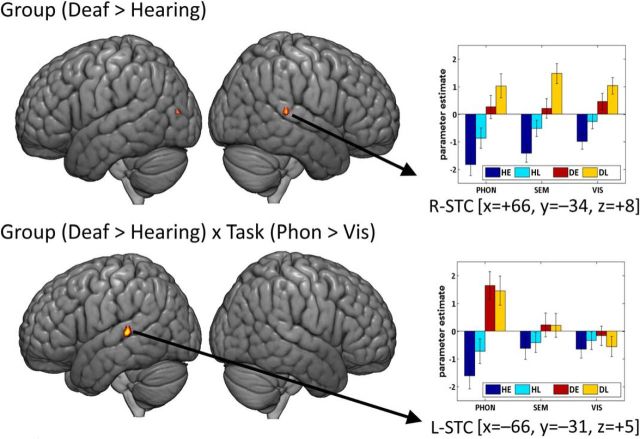
The main effect of deafness and the interaction of deafness and task at *p* < 0.05 FWE-corrected (red to yellow). At the FWE-corrected level, these effects in STC were task-independent on the right (top) and task-dependent on the left (bottom). The bar plots of parameter estimates at these peaks are also shown. Error bars indicate SE. PHON, Phonological task; SEM, semantic task; VIS, visual task.

There were group by task interactions in the left STC, significant at *p* < 0.05 FWE-corrected (for details, see Table 3). These indicated enhanced activation in deaf relative to hearing signers only for the BSL phonological task (Fig. 3). The location of the enhanced left STC activation was in the posterior superior temporal gyrus and sulcus and did not include Heschl's gyrus. Rather, activation was within the higher-order auditory cortex Te 3, defined by the SPM Anatomy Toolbox v2.2b (Eickhoff et al., 2005, 2006, 2007). Within the deaf participants, left STC activation was significantly greater for the BSL phonological task than the semantic task or the visual task. The difference in activation during the semantic and visual tasks was also significant (Table 3). The main effect of deafness, across the three tasks, was only significant in the left STC at the *p* < 0.001 uncorrected level (*x* = −66, *y* = −34, *z* = +5; *Z* = 3.55, *k* = 5).

**Table 3.** Statistical details for hearing status and task interactions in left STC

	Deaf > hearing					*k*		Z-score relative to baseline	
	x	y	z	Z-score	*p* value	FWE corrected	*p* < 0.001 uncorrected	Deaf	Hearing
BSL phonological task	−66	−31	+5	5.26	0.004 FWE	7	104	4.14*	−3.47*
Semantic task	−63	−34	+5	2.70	n.s.	N/A	N/A	1.79	−0.90
Visual task	−66	−28	+2	0.71	n.s.	N/A	N/A	−0.27	−2.49
BSL phonological > semantic	−66	−31	+5	5.80	<0.001 FWE	7	173	5.20**	−3.08
BSL phonological > visual	−66	−31	+5	5.93	<0.001 FWE	26	259	5.77**	−2.51
Semantic > visual	−63	−37	+2	3.56	<0.001 uncorr	N/A	22	6.16**	2.17

Also shown is the Z-score at the same peak for deaf participants relative to baseline; and hearing participants relative to baseline to illustrate their response.

* = significant at *p* < 0.001 uncorrected;

** = significant at *p* < 0.05 FWE corrected for multiple comparisons across the whole brain.

A very different response pattern was observed in the left STC in hearing signers. During the BSL phonological task, hearing signers showed deactivation, although this was only significant at the *p* < 0.001 uncorrected level (*x* = −66, *y* = −31, *z* = +5; *Z* =−3.47, *k* = 1104). Although deactivation for the BSL phonological task was numerically greater than the semantic task, which in turn was numerically greater than the visual task, there was no significant difference across tasks (Table 3).

There was no main effect of age of acquisition in left STC (*p* > 0.001 uncorrected). There were no significant age of acquisition by task interactions and no three-way interactions between age of acquisition, group, and task.

#### Right STC

Across tasks, the right STC showed significantly greater activation in the deaf than hearing signers, (*x* = +66, *y* = −34, *z* = +8; *Z* = 5.35, *p* = 0.002, *k* = 14 FWE-corrected). This task-independent effect of deafness in the right STC was observed in the homolog to the region showing a task-dependent effect of deafness in the left STC (Fig. 3).

There were no significant group by task interactions at *p* < 0.05 FWE-corrected. However, these interactions were present at a lower threshold of *p* < 0.001 uncorrected (Table 4). The effect of age of acquisition (late > early) in the right STC, was significant only at *p* < 0.001 uncorrected (*x* = +57, *y* = −34, *z* = +11; *Z* = 3.19, *k* = 3). Late learners showed greater activation (deaf) or reduced deactivation (hearing) than early learners. None of the interactions between age of acquisition and task; age of acquisition and group; or age of acquisition, group, and task reached significance (*p* > 0.001 uncorrected).

**Table 4.** Statistical details for hearing status and task interactions in right STC

	Deaf > hearing					*k*		Z-score relative to baseline	
	*x*	*y*	*z*	Z-score	*p* value	FWE corrected	*p* < 0.001 uncorrected	Deaf	Hearing
BSL phonological task	+66	−34	+8	4.69	<0.001 uncorr	N/A	150	2.12	−4.73*
Semantic task	+66	−34	+8	5.08	0.010 FWE	3	67	3.29*	−4.11*
Visual task	+66	−34	+8	4.63	<0.001 uncorr	N/A	37	3.49*	−3.18*
BSL phonological > semantic	+69	−28	−1	3.70	<0.001 uncorr	N/A	12	4.71*	−0.08
BSL phonological > visual	+60	−31	+2	3.99	<0.001 uncorr	N/A	20	3.22*	−2.46
Semantic > visual	+60	−28	+2	3.39	<0.001 uncorr	N/A	2	2.24	−2.66

Also shown is the Z-score at the same peak for deaf participants relative to baseline; and hearing participants relative to baseline to illustrate their response.

* = significant at *p* < 0.001 uncorrected.

#### Hemispheric differences

**Figure 4.**
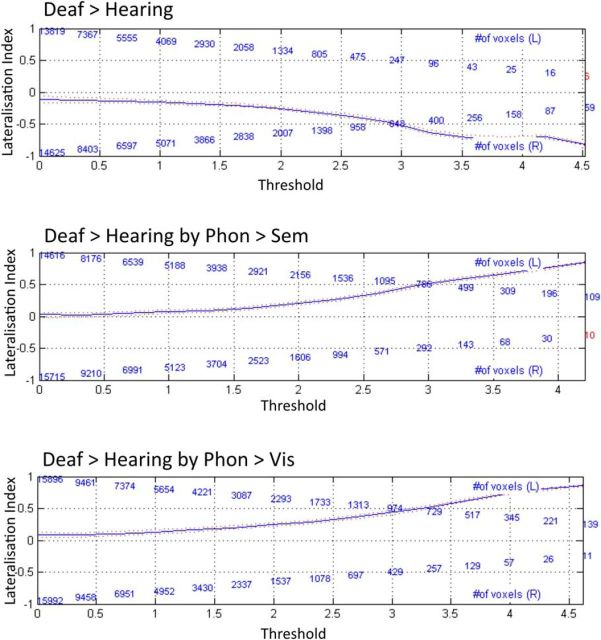
LI values for top (deaf > hearing), middle (deaf > hearing by the BSL phonological task > the semantic task), and bottom (deaf > hearing by the BSL phonological task > the visual task) within temporal cortices.

At the corrected level (*p* < 0.05 FWE), the data demonstrated significant group by task interactions in the left STC (deaf > hearing in the phonological task only) and a significant group effect in the right (deaf > hearing in all 3 tasks). However, assessing laterality effects is, among other things, dependent on the statistical threshold used. Indeed, at the lower threshold of *p* < 0.001 uncorrected, we found group by task interactions in the right STC and a main effect of group in the left STC. To determine whether auditory experience differentially influences the function of left and right STC regardless of statistical thresholds, we performed additional analyses to directly test for the hemispheric differences in STC. Bootstrapped laterality analyses (Wilke and Schmithorst, 2006; Wilke and Lidzba, 2007) confirmed that the main effect of group was right lateralized (weighted mean = −0.53; trimmed mean = −0.35), whereas both interaction effects involving group and task were left lateralized (phonological > semantic: weighted mean = 0.49, trimmed mean = 0.27; phonological > visual: weighted mean = 0.53, trimmed mean = 0.32). Lateralization index values are plotted in Figure 4.

#### Other regions

Deaf signers also showed greater activation than hearing signers, across all tasks, in visual processing regions (Table 5; Fig. 3) even though the stimuli, accuracy and response times did not differ for deaf and hearing participants. No regions were activated significantly more in hearing than deaf participants.

**Table 5.** Statistical details for the regions in which activation was greater for deaf than hearing signers across all tasks at *p* < 0.05 FWE-corrected (*Z* > 4.76)

Region	Deaf > hearing			*Z*-score	*p* value (FWE-corrected)	*k*		Z-score > baseline	
	*x*	*y*	*z*			FWE-corrected	*p* < 0.001 uncorrected	Deaf	Hearing
Temporal									
R									
Superior temporal gyrus	+66	−34	+8	5.35	0.002	6	86	3.27*	−4.55**
Occipital									
R									
Lingual gyrus	+21	−85	−4	5.11	0.008	1	29	8.25**	6.54**
R									
Middle occipital gyrus	+45	−79	−7	4.83	0.035	2	32	8.69**	8.08**
L									
Middle occipital gyrus	−30	−85	+11	5.22	0.005	4	56	8.71**	8.03**
L									
Fusiform gyrus	−33	−61	−7	5.05	0.011	1	21	8.04**	5.24**
L									
Calcarine sulcus	−12	−94	+2	4.84	0.033	2	25	8.61**	7.92**

Also shown is the Z-score at the same peak for deaf participants relative to baseline; and hearing participants relative to baseline to illustrate their response.

* = significant at *p* < 0.001 uncorrected;

** = significant at *p* < 0.05 FWE corrected for multiple comparisons across the whole brain.

### Summary

Deaf participants showed increased activation relative to hearing participants in both left and right STC. This effect was greatest during the BSL phonological task in left STC. In contrast, enhanced activation in the deaf group was not task dependent in the right STC. Analyses directly testing the hemispheric differences confirmed that the interaction of deafness and task was more left lateralized, whereas the main effect of deafness was more right lateralized.

## Discussion

Understanding how biological and environmental constraints influence neural plasticity is fundamental to a complete understanding of the brain. Unique insights into these questions can be gained from working with those who are born profoundly deaf. Unlike research with deaf animal models (Lomber et al., 2010; Kral et al., 2016), research with deaf humans must take into account the influence of accessing language primarily through the visual modality and the age of acquisition of that visuospatial language to fully understand experience-dependent neural plasticity (Campbell et al., 2014). Prior studies have shown that activation in the STC in response to sign language stimuli is significantly greater in deaf native signers than hearing native signers (MacSweeney et al., 2002, 2004). Here we investigated the functional role of the left and right STC in deaf signers by manipulating task demands and the age at which sign language was acquired.

Our results reveal that deaf and hearing signers show contrasting effects in the STC during BSL phonological decisions on pictures of objects. The region showing differential effects included the posterior superior temporal gyrus and sulcus but excluded Heschl's gyrus. Deaf signers showed STC activation, which was absent in hearing signers. These contrasting effects were observed even though the stimuli and task instructions were identical for all participants, and even though there was no significant difference in response times for the deaf and hearing participants, all of whom had similar sign language experience.

Our results also differentiate responses in the left and right STC. Specifically, left STC was more sensitive to task than deafness, whereas right STC was more sensitive to deafness regardless of task. We consider whether and how the left and right STC contribute to visual cognition, in those born deaf and in those born hearing.

### Left STC function in those born deaf

The task-dependent effects in left STC provide clues to its computational function. Activation increases were strongest when the demands on visual imagery and visuospatial working memory were highest. This observation (*x* = −66, *y* = −31, *z* = +5 in MNI space) is consistent with prior evidence that deaf participants show increased activation in the similar part of STC (*x* = −51, *y* = −33, *z* = +6 in MNI space) during the maintenance and recognition phases of a visuospatial working memory task with nonverbal stimuli (Ding et al., 2015). It also falls within the cytoarchitectonic region (Te 3) where Bola et al. (2017) found enhanced STC activation in deaf participants during a visual rhythm working memory task involving sequences of flashes. The contribution of left STC to visuospatial processing in deaf participants might therefore explain responses observed in response to both verbal and nonverbal stimuli. In hearing people, in addition to speech recognition and phonological processing (Hickok, 2009; Okada et al., 2010; Evans et al., 2014), this part of the left STC has been implicated in auditory working memory (Leff et al., 2009) and auditory imagery (McNorgan, 2012). Demonstrating the involvement of the left STC in visuospatial processing in those born deaf complements what has been observed in congenitally deaf cats. For example, Lomber et al. (2010) has shown that parts of auditory cortex that are usually involved in identifying auditory location in hearing cats are recruited to identify visual location in deaf cats, whereas regions involved in identifying auditory movement in hearing cats are recruited to process visual motion in deaf cats.

We found no evidence for the influence of age of acquisition in the left STC activation. At first glance, this may appear to be inconsistent with prior studies showing early sign language acquisition can improve nonverbal working memory (Marshall et al., 2015) and sign language processing, particularly grammaticality judgements (Mayberry et al., 2011; Cormier et al., 2012; Henner et al., 2016). Earlier sign language acquisition has also been reported to be related to increased left STC activation (Mayberry et al., 2011). However, the effect of age of acquisition on both behavior and brain activation is highly task-dependent. For example, Mayberry et al. (2011) did not see an advantage of early sign language acquisition in behavioral performance when their participants were engaged in a phonemic-hand judgment task, nor an effect on brain activation during passive viewing of a still image of the signer. In addition, age of acquisition is often correlated with proficiency. In our study, we matched the sign language proficiency across those who learnt sign language early versus late, and this might explain why left STC activation was not influenced by age of acquisition in our participants. Future studies will need to dissociate effects that are related to age of sign language exposure and, separately, to sign language proficiency.

### Left STC function in those born hearing

Although deaf signers showed enhanced left STC activation during the BSL phonological task relative to other tasks, hearing signers did not activate this region. This contrasting pattern was observed even though they had the same sign language experience and performance.

We propose that our hearing participants may have been suppressing distracting auditory information from the environment. Indeed, deactivation in sensory cortices when attending to another sensory input is a well-documented phenomena (Laurienti et al., 2002; but see Ding et al., 2015). For example, hearing non-signers have been shown to deactivate STC when performing a visual rhythm task (Bola et al., 2017) and also a visual imagery task (Zvyagintsev et al., 2013). Participants have also been shown to deactivate visual cortex while performing auditory spatial and pitch judgment tasks (Collignon et al., 2011). This modality-specific deactivation allows the down regulation of potentially distracting sensory activity in other modalities, for example, scanner noise in hearing participants doing a visually demanding task. Although deactivation in hearing signers in the current study did not reach the threshold for statistical significance a similar explanation may explain the pattern observed in this group.

It is interesting that although hearing signers in the current study and hearing non-signers in Bola et al. (2017) did not activate the STC, hearing non-signers tested by Ding et al. (2015) showed positive activation. The potential cause of the discrepancy in STC deactivation in hearing participants between studies is unclear and requires investigation.

### Right STC function in those born deaf and those born hearing

Unlike the left STC, deaf participants activated right STC regardless of the task demands. Activation is therefore more likely to reflect bottom up, perceptual processing of visual stimuli than linguistic processing or visuospatial imagery or working memory demands. This is consistent with prior literature showing deafness related increases in right STC activation to a range of nonverbal visual stimuli such as moving dot arrays (Finney et al., 2001; Fine et al., 2005; Vachon et al., 2013) and static and moving sinusoidal gratings (Shiell et al., 2014). In contrast, hearing participants did not activate STC in response to any of the tasks.

There was also a main effect of age of sign language acquisition in the right STC (late > early). However, this had not been predicted and was significant only at an uncorrected level. Further studies are necessary to examine this potential effect.

### Hemispheric differences in STC in deaf signers

Finally, we found that the main effect of group was right lateralized, with deaf signers demonstrating significantly greater activation than hearing signers. In contrast, interactions of group and task (deaf > hearing by BSL phonological task > semantic task; deaf > hearing by BSL phonological task > visual task) were left lateralized. These hemispheric differences were not reported in the Bola et al. (2017) study and only reported during the encoding phase of a visual memory task in the Ding et al. (2015) study. Because neither study used linguistic stimuli, it is likely that the hemispheric differences identified in the current study reflect the additional contribution of the left STC to the increased visuospatial processing demands of the BSL phonological task.

### Conclusions

Together our results from deaf and hearing signers suggest that the function of posterior STC, which includes the posterior superior temporal gyrus and sulcus but excludes Heschl's gyrus, changes with auditory experience. In those born hearing, left and right STC primarily responds to auditory stimuli and is suppressed, to some extent, during visual tasks. In contrast, when the STCs do not receive auditory input, left STC participates in cognitive tasks including those that require visuospatial processing and right STC participates in low-level visual processing, regardless of visuospatial demands. As all our participants were proficient signers, future studies are now required to determine how sign language knowledge and importantly, sign language proficiency, influence the strong effect of deafness on visuospatial processing in STCs that we have described here.
